# Development of a new Rasch-based scoring algorithm for the National Eye Institute Visual Functioning Questionnaire to improve its interpretability

**DOI:** 10.1186/s12955-017-0726-5

**Published:** 2017-08-14

**Authors:** Jennifer Petrillo, Neil M. Bressler, Ecosse Lamoureux, Alberto Ferreira, Stefan Cano

**Affiliations:** 1 Biogen, Boston, USA; 2 Retina Division, Wilmer Eye Institute, Johns Hopkins University School of Medicine, Baltimore, MD USA; 3 Centre for Eye Research Australia, the Royal Victorian Eye and Ear Hospital, University of Melbourne, Melbourne, VIC Australia; 4 Singapore Eye Research Institute, Singapore, Singapore; 5 Novartis Pharma AG, CH-4002 Basel, Switzerland; 6 Modus Outcomes, Letchworth Garden City, UK

**Keywords:** National Eye Institute Visual Functioning Questionnaire, Rasch Measurement Theory, Vision-related quality of life

## Abstract

**Background:**

The NEI VFQ-25 has undergone psychometric evaluation in patients with varying ocular conditions and the general population. However, important limitations which may affect the interpretation of clinical trial results have been previously identified, such as concerns with reliability and validity. The purpose of this study was to evaluate the National Eye Institute Visual Functioning Questionnaire (NEI VFQ­25) and make recommendations for a revised scoring structure, with a view to improving its psychometric performance and interpretability.

**Methods:**

Rasch Measurement Theory analyses were conducted in two stages using pooled baseline NEI VFQ­25 data for 2487 participants with retinal diseases enrolled in six clinical trials. In stage 1, we examined: scale-to-sample targeting; thresholds for item response options; item fit statistics; stability; local dependence; and reliability. In stage 2, a post-hoc revision of the scoring structure (VFQ-28R) was created and psychometrically re-evaluated.

**Results:**

In stage 1, we found that the NEI VFQ­25 was mis-targeted to the sample, and had disordered response thresholds (15/25 items) and mis-fitting items (8/25 items). However, items appeared to be stable (differential item functioning for three items), have minimal item dependency (one pair of items) and good reliability (person-separation index, 0.93). In stage 2, the modified Rasch-scored NEI VFQ­28­R was assessed. It comprised two broad domains: Activity Limitation (19 items) and Socio-Emotional Functioning (nine items). The NEI VFQ­28­R demonstrated improved performance with fewer disordered response thresholds (no items), less item misfit (three items) and improved population targeting (reduced ceiling effect) compared with the NEI VFQ­25.

**Conclusions:**

Compared with the original version, the proposed NEI VFQ­28­R, with Rasch-based scoring and a two-domain structure, appears to offer improved psychometric performance and interpretability of the vision-related quality of life scale for the population analysed.

## Background

The 25-item National Eye Institute Visual Functioning Questionnaire (NEI VFQ­25) is a patient-reported outcome (PRO) instrument originally developed for use in patients with age-related macular degeneration (AMD), cataracts, diabetic neuropathy and glaucoma [1, 2]. It has been widely used in clinical trials in neovascular AMD [3, 4], diabetic macular edema (DME) [5, 6], macular edema due to retinal vein occlusion (RVO) [7] and choroidal neovascularization (CNV) secondary to pathologic myopia (PM) [8].

When using PROs in clinical studies, it is critical that the instrument selected provides a valid measurement of the concept of interest in the specific context of use [9–11]; this has become especially relevant in recent years because the use of data from PRO instruments, such as the NEI VFQ-25, in decisions about healthcare resource allocation is increasing [12–14]. The NEI VFQ-25 has undergone psychometric evaluation in patients with varying ocular conditions and the general population [15–20]. However, important limitations have been identified which may affect the interpretation of clinical trial results based on the NEI VFQ-25: for example concerns with reliability and validity [16, 17, 19, 20], as well as the dimensional structure of the NEI VFQ-25 validity [16, 17, 19, 20]. Furthermore, a more complete understanding of the content, clinical validity, and interpretability of the NEI VFQ­25 is likely to be critical to regulatory acceptance of PRO-based labelling claims for new drugs and devices [7, 21].

Classical test theory is associated with four key challenges: first, the analysis is framed in ordered counts, not interval-level measurement; second, findings are both sample and scale dependent; third, missing data cannot be handled easily; and fourth, the standard error of measurement around individual patients’ scores is assumed to be a constant value regardless of the person’s location on the range of a scale [22–24]. Modern psychometric methods, such as Rasch Measurement Theory (RMT), provide a more robust approach with which to examine issues such as validity and interpretability compared with traditional psychometric methods [22–24]. Rasch analysis has previously been used to “re-engineer” the NEI VFQ-25 scale to comprise two valid and unidimensional subscales, namely visual functioning and emotional well-being [16, 17, 19, 20].

This study uses a large and well-described patient population to extend previously published research proposals for a two-scale structure to further our understanding of how the NEI VFQ-25 can: 1) capture the patient perspective and include clinically relevant and meaningful domains; through 2) exploiting the benefits of Rasch Measurement Theory, and in particular item maps and threshold plots to improve interpretability; and ultimately 3) provide a scoring algorithm that can ensure an equivalent frame of reference across different clinical settings for patients with retinal diseases.

## Methods

### Study population

This post-hoc data analysis was conducted on pooled baseline NEI VFQ-­25 data for 2487 participants (mean [SD] age, 64 [90] years; range, 18–96 years; 53% men) from six clinical trials investigating the efficacy of ranibizumab treatment in patients with visual impairment due to neovascular AMD, DME, macular edema due to RVO, or CNV secondary to PM (Table 1) [5, 8, 25–28]. The studies included patients with a broad geographic distribution, including patients from US, Canada, Australia, Japan as well as several European and Asian countries (Table 1).

**Table 1:** Study dataset summary and key baseline patient characteristics

Indication	Trial	*n* (%)	Age (years) mean (SD, range)	BCVA letter score at baseline mean (SD, range), Snellen equivalents
DME	RESTORE [5]	344 (13.8)	64 (9, 37–87)	73 (11, 20/32) 96–40 (20/12–20/160)
Macular edema due to branch RVO	BRAVO [28]	392 (15.8)	66 (12, 26–91)	82 (11, 20/25) 99–29 (20/10–20/250)
Macular edema due to central RVO	CRUISE [27]	385 (15.5)	68 (13, 20–91)	81 (11, 20/25) 100–29 (20/10–20/250)
Neovascular AMD	ANCHOR [25]	418 (16.8)	77 (8, 53–96)	68 (18, 20/40) 100–2 (20/10–20/1000)
Neovascular AMD	MARINA [26]	716 (28.8)	77 (7, 52–95)	68 (15, 20/40) 99–19 (20/10–20/400)
CNV secondary to PM	RADIANCE [8]	232 (9.3)	56 (14, 18–87)	73 (13, 20/32) 95–30 (20/12–20/250)
Overall population		2487 (100)	64 (9, 18–96)	73 (15, 20/32) 100–2 (20/10–20/1000)

*AMD* age-related macular degeneration, *BCVA* best-corrected visual acuity, *BSE* better-seeing eye, *CNV* choroidal neovascularization, *DME* diabetic macular edema, *PM* pathologic myopia, *RVO* retinal vein occlusion, *SD* standard deviation, *VEGF* vascular endothelial growth factor

### NEI VFQ­-25

The NEI VFQ­-25 is comprised of: one general health item (VF1) and 24 items (VF2 to VF25) that assess visual functioning and the impact of vision problems on physical and social functioning and emotional well-being [2]. The vision-related items are grouped into 11 sub-domains (general vision, ocular pain, near activities, distance activities, social function, mental health, role difficulties, dependency, driving, colour vision, peripheral vision) including one to four items each. The NEI VFQ­-25 Appendix of Optional Additional Questions includes extra items that can be added to specific subscales. Responses to Optional Additional Questions associated with the near and distance activities subscales (VFA3 to VFA8) were available for four of the six studies [5, 8, 25–28]. These were included in this analysis. Table 2 shows a list of items, item codes and summary statements used in this for reference throughout the article; question VH1 was excluded from the analysis as it refers to general health and is not vision-specific.

**Table 2:** NEI VFQ-25 Item Codes and Summary Statements and Additional Items Used in the Construction of the NEI VFQ-28- R

Item	Statement Summary	NEI VFQ-25 (Reliability: 0.93)					NEI VFQ-28-R (Reliability: 0.94)				
		D^r^	M^r^	M^t^	D^f^	D^p^	D^r^	M^r^	M^t^	D^f^	D^p^
VF2	Eyesight quality	✗	✓	✓	✓	✓	✓	✓	✓	✓	✓
VF3	Time spent worrying about eyesight	✗	✗	✗	✗	✓	✓	✓	✓	✓	✓
VF4	Amount of pain in/around eyes	✗	✗	✗	✓	p1	✓	✓	✓	✓	✓
VF5	Difficulty reading newspaper print size	✓	✓	✓	✓	✓	✓	✓	✓	✓	p1
VF6	Difficulty seeing well up close	✓	✓	✓	✓	✓	✓	✓	✓	✓	✓
VF7	Difficulty finding objects	✓	✗	✓	✓	✓	✓	✓	✓	✓	✓
VF8	Difficulty reading street signs	✓	✓	✓	✓	✓	✓	✓	✓	✓	✓
VF9	Difficulty going downstairs or curbs in dim light	✓	✓	✓	✗	✓	✓	~	✓	~	✓
VF10	Difficulty with peripheral vision	✓	✓	✓	✓	✓	✓	✓	✓	~	✓
VF11	Difficulty seeing other people reactions	✓	✗	✓	✓	✓	✓	✓	✓	✓	✓
VF12	Difficulty matching clothes	✗	✓	✓	✓	✓	✓	✓	✓	✓	✓
VF13	Difficulty visiting others	✗	✗	✓	✓	✓	✓	~	✓	✓	✓
VF14	Difficulty going out	✗	✗	✓	✓	✓	✓	✓	✓	✓	✓
VF15C	Difficulty driving during day	✗	✓	✓	✓	✓	✓	✓	✓	✓	✓
VF16	Difficulty driving at night	✗	✓	✓	✗	✓	✓	✓	✓	~	p2
VF16A	Difficulty driving in difficult conditions	✗	✓	✓	✓	✓	✓	✓	✓	✓	p2
VF17	Limited in time to accomplish activities	✓	✓	✓	✓	✓	✓	✓	✓	✓	✓
VF18	Limited in time at work due to eyesight	✓	✓	✓	✓	✓	✓	✓	✓	✓	✓
VF19	Limited in time due to pain or discomfort around eyes	✓	✓	✗	✓	p1	✓	✓	✓	✓	✓
VF20	Time staying at home due to eyesight	✓	✓	✓	✓	✓	✓	✓	✓	✓	✓
VF21	Time frustrated due to eyesight	✓	✗	✗	✓	✓	✓	✓	✓	✓	✓
VF22	Amount of control lost due to eyesight	✓	✓	✓	✓	✓	✓	✓	✓	✓	✓
VF23	Reliance on what other people say due to eyesight	✓	✗	✓	✓	✓	✓	✓	✓	✓	p3
VF24	Amount of help from others due to eyesight	✓	✓	✓	✓	✓	✓	~	✓	✓	p3
VF25	Embarrassed doing things because of eyesight	✗	✓	✓	✓	✓	✓	✓	✓	✓	✓
VFA3	Difficulty reading small print (e.g. medicine bottle)	N/A	N/A	N/A	N/A	N/A	✓	✓	✓	✓	p1
VFA4	Difficulty reading mail/bills accurately	N/A	N/A	N/A	N/A	N/A	✓	✓	✓	✓	✓
VFA5	Difficulty shaving or putting on makeup	N/A	N/A	N/A	N/A	N/A	✓	✓	✓	~	✓
VFA6	Difficulty recognizing faces due to eyesight	N/A	N/A	N/A	N/A	N/A	✓	✓	✓	✓	✓
VFA7	Difficulty taking part in outdoor activities	N/A	N/A	N/A	N/A	N/A	✓	✓	✓	✓	✓
VFA8	Difficulty seeing television	N/A	N/A	N/A	N/A	N/A	✓	✓	✓	✓	✓

D^r^: Items with disordered response thresholds

M^r^: Mis-fitting items (based on fit Residuals outside −2.5 to +2.5)

M^t^: Mis-fitting items (based on statistically significant Item–trait chi-squared values)

D^f^: Significant differential item functioning (DIF), based on statistically significant F-values

D^p^: Item pairs with potential dependency (*r* > 0,30)

Most individual items are scored by respondents using a 5- or 6-point response scale, ranging from (1) ‘not affected at all’, to (4) ‘severely affected’, (5) ‘stopped doing this because of my eyesight’ and (6) ‘stopped doing this for other reasons’. True/false items are scored on a 5-point response scale, ranging from (1) ‘definitely true’ to (5) ‘definitely false’, with (3) indicating ‘not sure’. Responses for each item are converted to a score between 0 and 100; high scores represent better visual functioning than low scores. Subscale scores are calculated as the mean of all component item scores. An overall composite score is calculated as the mean of all 11 sub-domain scores, and is assumed to be a unidimensional scale measuring vision-related quality of life (QoL) [2].

### Rasch measurement theory

The field of psychometrics is concerned with evaluation of the measurement properties (e.g. reliability, validity, ability to detect change) of scales and tests [29]. Traditional psychometric methods have important limitations that are overcome by modern methods [22, 23]. RMT is used in the current study [30, 23]. RMT analysis indicates the extent to which rigorous measurement is achieved by examining the difference (or ‘fit’) between the observed scores (patients’ responses to items) and the expected values predicted from the data by the Rasch model [30, 31]. A range of evidence is used to evaluate each individual item in the scale and make a judgment about the overall quality of the scale. These methods are increasingly used in health outcomes research [22, 32, 33], and have previously been applied to the NEI VFQ­25 [18, 34, 35].

There were two stages of analysis: 1) evaluation of the measurement performance of the NEI VFQ-25 using RMT; and 2) exploration of the potential for an alternate scoring structure based on previous research [17, 18, 36], followed by an empirical post-hoc analysis of this structure including provision of how to interpret the proposed transformed scoring structure.

### Stage 1: RMT analysis of the NEI VFQ-25

RMT analysis, based on the unrestricted Rasch Model for polytomous ordered responses, was performed on the NEI VFQ-25 using RUMM2030 software (RUMM Laboratory Pty Ltd., Perth, WA, Australia) [37]. For this analysis, we focused on the complete NEI VFQ-25 item set as opposed to the individual sub-domains. Results were interpreted with reference to published criteria wherever possible. There were six areas of evaluation: scale-to-sample targeting; threshold for item response options; item fit statistics; stability; local dependence; and reliability. These are presented in more detail, including references for criteria used, elsewhere [22] and summarized below.

Scale-to-Sample Targeting: The items of the NEI VFQ-25 should be targeted to the patient population under study, in this case patients with visual impairment due to neovascular AMD, DME, macular edema due to RVO, or CNV secondary to PM. Targeting is examined by inspecting the spread of person locations (i.e., range of vision-related QoL reported by the sample) and item locations (i.e., range of the vision-related QoL measured by the items in a scale). Items of the NEI VFQ-25 should be evenly spread across a reasonable ability range that matches the range of the vision-related QoL experienced by the patient sample.

Threshold for Item Response Options: The response categories for the NEI VFQ-25 were examined to determine if successive integer scores, which imply a continuum, increased for the vision-related QoL measured. We examined the ordering of thresholds, which are the points of crossover between adjacent response categories (e.g., between “Most of the Time” and “Some of the Time”).

Item Fit Statistics: We examined three indicators of fit to determine if the items work together to map out a vision-related QoL: (1) log residuals (item–person interaction); (2) Chi-square values (item–trait interaction); and (3) item characteristic curves (ICC). As a guide, the criteria for fit residuals should fall between −2.5 and +2.5. The Chi-square value for each item should be non-significant after Bonferroni adjustment.

Stability: Differential item functioning (DIF) measures the degree to which item performance remains stable across subgroups. A Chi-square value significant after Bonferroni adjustment can indicate an item with potential DIF. We examined DIF by different countries, studies, sex, visual acuity (BCVA) of the study eye, and treatment regimens.

Local Dependence: Residual correlations between items in a scale can artificially inflate reliability. There are different preferred criteria for cut-offs for residual correlations between items [38–40]. We selected <0.30 as this criterion represents 10% of the shared variance and is the currently most widely used in RUMM 2030 [41].

Reliability: We examined reliability using the Person separation index (PSI), a statistic that is comparable to Cronbach’s alpha. The PSI measures error associated with the measurement of people in a sample. High values indicate better reliability than low values.

### Stage 2: Construction and RMT analysis of the NEI VFQ-28-R

There were three steps to *Stage 2*: (1) review of findings from *Stage 1* and the conceptual content of the NEI VFQ-25 items; (2) re-structuring of the conceptual and measurement model of NEI VFQ-25 based on the empirical findings from Stage 1 and previously proposed conceptual framework (two domains – 19-item Activity Limitation and 9-item Socio-emotional Functioning) [17, 18, 36]; (3) analysis of the psychometric properties (as described in *Stage 1*) of the revised NEI VFQ-28-R scoring structure and comparison against the original.

## Results

### Stage 1: RMT analysis of the NEI VFQ

**Fig. 1:**
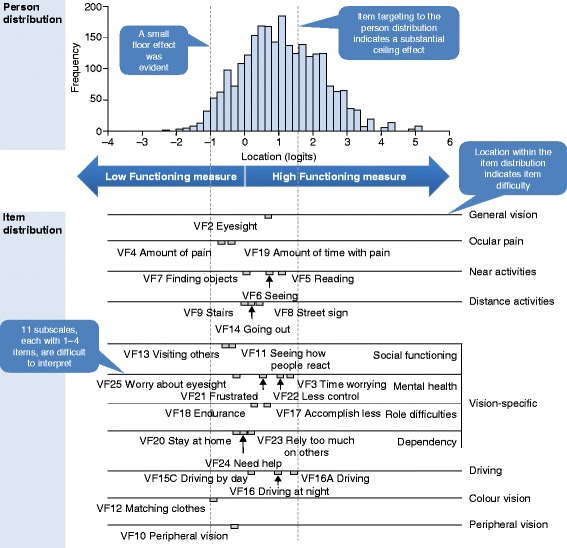
Scale-To-Sample (Person–Item) Distribution for the NEI VFQ­-25. The *top panel* shows the distribution of pooled participants on the visual functioning scale. The *bottom panel* maps the NEI VFQ-25 items (grouped by subscale) onto the same visual functioning scale, highlighting item difficulty. *Vertical dashed lines* indicate the lower (*left*) and upper (*right*) extent of instrument coverage for the pooled participant population

The psychometric analysis of the NEI VFQ­25 revealed mixed performance (summarized in Table 2, Fig. 1). Scale-to-sample targeting indicated a substantial ceiling effect, with few items in the NEI VFQ­-25 measuring differences in vision related QoL among study participants with better levels of visual ability (Fig. 1). Furthermore, all 11 of the NEI VFQ-­25 subscales contained small numbers of items and measured only very limited ranges of vision related QoL (Fig. 1). Fifteen of the 25 items had disordered item-response thresholds, suggesting a problem with either the number or type of response option in each instance. Analysis of item fit validity showed: eight items had residuals outside the range of −2.5 to +2.5; four items had statistically significant item–trait chi-squared values; and based on ICCs, the greatest deviations from the Rasch model were for items VF3, VF4, VF16, VF19 and VF21. However, there was minimal item dependency, with a residual correlation greater than 0.30 between only one pair of items, minimal DIF (except VF3, DIF by study; VF9 and VF16, DIF by gender), and reliability was good (estimated PSI, 0.93).

### Stage 2: Construction and RMT analysis of the NEI VFQ-28-R

**Fig. 2:**
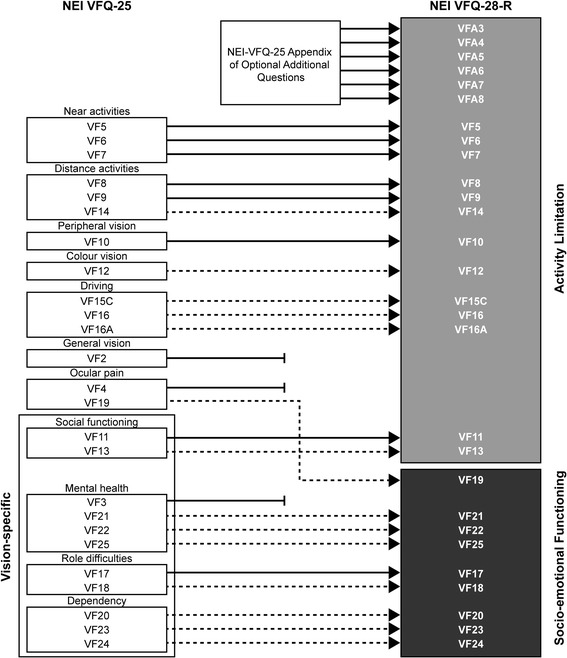
Development of the NEI VFQ-­28­-R. Items retained from the unchanged NEI VFQ-25, and items from the NEI VFQ-25 Appendix, are indicated by *solid arrows*. NEI VFQ-25 items excluded from the NEI VFQ-28-R are indicated by *terminated lines*. NEI VFQ-25 items that had their response levels modified (response levels combined or one level rescored as missing data) are indicated by *dashed arrows*

Based on the results of the RMT analysis, several modifications were tested to improve the instrument through revisions to the item set and scoring method (further details available from authors). In brief, three mis-fitting items were excluded (VF2, VF3, VF4), and six items were added (three near vision activity and three distance vision activity items; VFA3–8) from the NEI VFQ-­25 Appendix of Optional Additional Questions. Item response levels were combined for nine items with disordered response thresholds (VF12–14, VF15C, VF16, VF16A, VF18, VF19 and VF25), and five ‘true/false’ items had the ‘not sure’ response level rescored as missing data (VF20–24). The remaining 28 items were evaluated to fit within the NEI VFQ-28-R (Rasch-scored version) two-domains: Activity Limitation and Socio-Emotional Functioning (Fig. 2).

**Fig. 3:**
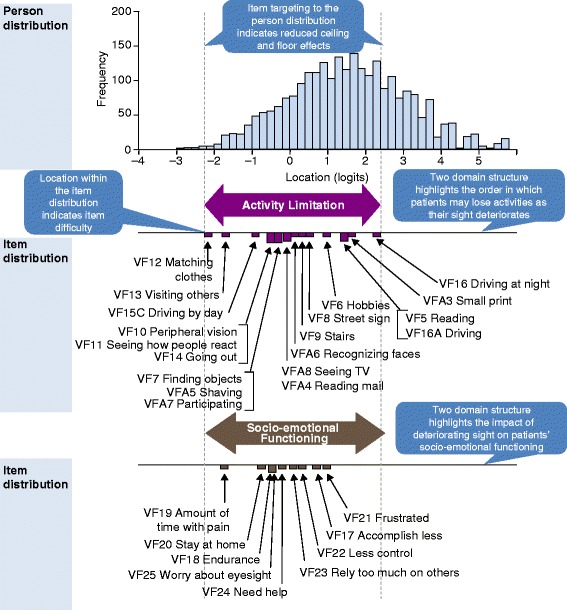
Scale-To-Sample (Person–Item) Distribution for the NEI VFQ-­28­-R. The *top panel* shows the distribution of pooled participants on the visual functioning scale. The *bottom panel* maps the NEI VFQ-28-R items (grouped into two domains) onto the same visual functioning scale, highlighting item difficulty. *Vertical dashed lines* indicate the lower (*left*) and upper (*right*) extent of instrument coverage for the pooled participant population

The NEI VFQ-­28-­R showed improved scale-to sample targeting (Fig. 3), threshold ordering and item fit compared with the NEI VFQ­-25 (Table 2). The two proposed NEI VFQ-­28-­R domains measure activity limitation and socio-emotional impact over a wider range of visual functioning (Range: −2.25 to 2.25 logits; Fig. 3) than any of the 11 individual NEI VFQ­-25 sub-domains (Range: −1 to 1.5 logits; Fig. 1).

## Discussion

Our psychometric evaluation of the NEI VFQ-­25, which supports previous research [17–19], suggests that the instrument can be improved as a measure of vision-related QoL. Importantly, by using RMT analysis, our findings provided a direct evidence-base upon which to propose a modified scoring system (NEI VFQ-­28-­R), which subsequently demonstrated improved psychometric performance. Furthermore, compared with the original NEI VFQ-25, the two-domain structure of the NEI VFQ-­28-­R measures activity limitation and socio-emotional impact over a wider range of visual functioning than the original 11-sub-domains of the NEI VFQ-25. Our analyses identified the same item misfit and threshold disorder as previous Rasch analyses. This suggests that previously reported limitations of the NEI VFQ-25 were not sample- or analysis-dependent, and warranted further recommendations to improve the validity of the instrument.

The Rasch-based scoring of the NEI VFQ-­28­-R places items and participants on the same linear scale of vision-related QoL. The location of participants on the scale indicates the impact of vision problems on their QoL, while the location of items indicates the perceived difficulty of activities for participants. This provides a better understanding of the measurement scale and how it relates to the range of visual functioning in the study population at an individual or group-level, than that provided by the original NEI VFQ-25 scoring conventions. In this paper, we defined interpretability in the context of exploiting the clinical hierarchy of the item ordering in the Rasch item map (and ultimately subsequent threshold plots) to define and describe the meaning of total sub-scale scores. With the items now on a continuous scale which matches the sample ability, score changes can be interpreted as specific functioning or well-being lost or gained. A comparison of scores can then be linked to specific ability of the patients.

The modified instrument, therefore, enables the identification of specific activities likely to be affected in patients with a known level of visual functioning as their vision improves or deteriorates. For example, on average, a patient with a high level of visual functioning experiencing a reduction in score as a result of progressive visual impairment will probably experience an impact on their ability to drive at night. Further deterioration in visual functioning may impact the patient’s ability to participate in hobbies that require them to see well up-close and may increase their need for help from others. Similarly, a patient with poor visual functioning experiencing improvements in vision as a result of treatment may become better able to go out to see movies, plays or sports events, and is likely to have a reduced need for help from others. This type of information is potentially of great value to clinicians in describing probable impacts on vision-related activities and socio-emotional functioning, and in guiding patient expectations regarding disease progression or treatment benefits.

It is important to highlight that while the psychometric performance and clinical interpretability of the NEI VFQ-­28­-R was improved compared with the NEI VFQ­25, scale-­to-­sample targeting indicated that a ceiling effect was still present. As such, the standard error associated with person estimates is lowest at the less impacted end of the continuum for both the NEIVFQ-28-R and the NEI VFQ-25 (around 0.2 logits; further information available from authors), respectively. This suggests the items associated with the lowest random error, and therefore most potential precision focus include core daily functioning (e.g., difficulty participating, shaving/styling, going down stairs), perceiving the environment (e.g., difficulty recognizing faces, peripheral vision, reading mail/bills, seeing television, reading street signs, finding objects), and burden (e.g., need help, reliance on others, needing to stay at home). However, the persistent ceiling effect means that the NEI VFQ-­28­-R may be unable to discriminate between participants with the highest levels of visual functioning.

Analysis of scale-­to-­sample targeting for the NEI VFQ­-28­-R among participant subgroups revealed that targeting to the scale was substantially better for participants with poorer visual acuity in the better-seeing eye (Early Treatment Diabetic Retinopathy Study [ETDRS] letter score, ≤ 58; approximate Snellen equivalent, 20/80 or worse) than for those with better visual acuity. This limitation may be addressed by adding items to the higher end of the visual functioning scale, but is an important consideration for comparisons of clinical trials in which the baseline visual acuity of the patient populations differs. Change from baseline assessments may be misleading, as a change from a ceiling score may not be feasible, regardless of the associated clinical benefit. Once again, importantly, by using RMT analysis, our findings provided a direct evidence-base upon which to attempt to identify items most relevant to patients with higher visual functioning. It is important to highlight that the item maps presented in this paper are the mapped item locations, not item thresholds. The threshold locations are more spread than the item locations (item location is a mean of item thresholds), and so ultimately it would be important to take these mapped item locations into consideration when interpreting the total scores from the two proposed sub-scales.

Our findings demonstrate that it is important to assess the psychometric properties of patient-reported outcome measures in each population to ensure they are reliable and valid for each specific population. This can be thought of as a quality control or calibration process (similar to calibrating scales to measure weight or a sphygmomanometer to measure blood pressure) whereby the measurement tool is checked for validity before the results are analysed so as to ensure accurate and precise measurement to reduce systematic bias [17–19].

Many studies have utilized Rasch analysis to optimize the psychometric properties of questionnaires. For example, the Impact of Vision Impairment questionnaire (IVI) was developed using classical test theory methods and originally comprised 32 items with five subscales [17–19]. Thorough re-examination using Rasch analysis demonstrated that the IVI’s most optimal structure was 28 items in three subscales, and a recent study has used Rasch techniques to shorten the scale further into 15 items in two subscales. Consequently, it is not uncommon for scales to be modified after undergoing additional validation in specific population samples; in fact, this serves to improve measurement precision and increase robustness of subsequent parametric testing using the questionnaire scores.

Our reengineering of the NEI VFQ does not have implications for other work which has used the NEI VFQ-25 to develop a utility measure from the NEI VFQ-25 items [18, 34, 35], as questionnaires and utility instruments are quite separate instruments with separate purposes, development processes and analysis requirements. We recommend to administer the NEI VFQ-25 items in full (including additional questions, and without modifications to the scale) to patients. This consistency in administration will allow improved comparisons of the measure to other studies, and for use in other purposes such as the VFQ-UI or other utility measures derived from these items. Additionally, our findings may inform future studies using the NEI VFQ-25 about the importance of assessing its psychometric properties in each population sample and by giving an a priori indication of its likely dimensional structure.

Finally, our study has two main limitations. First, it is a retrospective analysis of existing clinical trial data including patients diagnosed with retinal disease. Additional prospective evaluation will be required to establish the performance of the NEI VFQ­-28-­R in this patient group and those diagnosed with a cataract or other conditions associated with impaired vision, to establish the replicability and generalizability of our findings. Second, the two-domain structure (Activity Limitation and Socio-emotional Functioning) was proposed based on previous studies [18, 36]. In addition, the item hierarchies are empirically produced. However, this scoring structure proposes just one way that the items could be scored. The structure will require further consideration, qualitative research and clinical anchoring. In relation to this, it is important to flag that unidimensionality [42] is an important element of any Rasch analysis. However, dimensionality is a complex idea [43], made further complicated by the original NEI VFQ-25 was not developed with modern test theory principles in mind. [2] Thus, for this exploratory psychometric analysis [44], took recourse to the conceptual framework of the original authors [2] (which suggests for a single score) and the subsequent research supporting the two sub-scales structure [17].

## Conclusions

In summary, for patients with retinal diseases, the proposed NEI VFQ-­28­-R, which has Rasch-based scoring and a two-domain structure, provides improved psychometric performance and clinical interpretability relative to the original version. This Rasch-based approach provides an opportunity to move beyond working with raw scores to using instruments in a way that could facilitate item-level interpretation. Combined with the grouping of items into two clinically meaningful domains, the Rasch-based scoring in this revised instrument may allow identification of the probable impact of visual impairment on patients’ activity and socio-emotional functioning, helping to guide patient expectations.
